# Usefulness of bioelectrical impedance analysis and ECW ratio as a guidance for fluid management in critically ill patients after operation

**DOI:** 10.1038/s41598-021-91819-7

**Published:** 2021-06-09

**Authors:** Yoon Ji Chung, Eun Young Kim

**Affiliations:** 1 Department of Surgery, Seoul St. Mary’s Hospital, College of Medicine, The Catholic University of Korea, Seoul, South Korea; 2 Division of Trauma and Surgical Critical Care, Department of Surgery, Seoul St. Mary’s Hospital, College of Medicine, The Catholic University of Korea, Banpo-daero 222, Seocho-gu, Seoul, 137-701 South Korea

**Keywords:** Biomarkers, Risk factors

## Abstract

We determined the relationship between changes in bioelectrical impedance analysis (BIA) parameters and response of critically ill patients to fluid therapy during early postoperative period. Associations between BIA values indicating volume status of postoperative patient and clinical outcomes were also evaluated. From May 2019 to April 2020, patients who were admitted to the surgical intensive care unit (SICU) of our institution at more than 48 h after surgery were enrolled. Volume status was measured with a portable BIA device every morning for five days from SICU admission. Overhydration was defined as the case where extracellular water (ECW) ratio > 0.390 measured by BIA. Participants were daily classified into an overhydration or a normohydration group. The relationship between daily hydration status and postoperative outcome was evaluated. Most of the 190 participants showed the overhydration status in the first 48 h after surgery. The overhydration status on day 3 was significant predictor of postoperative morbidities (OR 1.182) and in-hospital mortality (OR 2.040). SOFA score was significant factor of postoperative morbidities (OR 1.163) and in-hospital mortality (OR 3.151) except for the overhydration status on day 3. Cut-off values of overhydration status by ECW ratio at day 3 for predicting postoperative morbidities and in-hospital mortality were > 0.3985 and > 0.4145, respectively. BIA would be a useful and convenient tool to assess the volume status of patients requiring intensive fluid resuscitation in early postoperative period. Overhydration status by ECW ratio on postoperative day 3 needs careful monitoring and appropriate interventions to improve clinical outcomes.

## Introduction

Critically ill patients in surgical intensive care unit (SICU) undergoing major operations have a wide range of organ injuries, vascular structural destruction, and extensive tissue removal that can lead to severe systemic inflammatory reactions and severe physiological stress. In early postoperative period, fluid resuscitation is performed to correct hemodynamic instability and compensate for severe fluid depletion. To estimate the adequacy of fluid therapy, various parameters such as serum lactate, venous saturation, and daily fluid balance have been used under clinical settings^1–3^. Unfortunately, these parameters have limitations in ease of use and accuracy^4–6^. Actually, most surgeons still perform fluid therapy after initial resuscitation based on clinician’s judgment and experience, and there is no guidance for optimal fluid management after initial resuscitation of major operations.

Bioelectrical impedance analysis (BIA) is a device that measures impedance such as resistance value caused by difference in electrical conductivity of each type of biological tissue such as fat and muscle. Electrical conductivity is proportional to the amount of water or electrolyte. This allows BIA to quantitatively measure the amount of water in and out of cells as well as fat and muscle mass and also to assess body composition status. Many previous studies have reported that BIA can be useful for assessing the prognosis of critical ill patients^4,5,7,8^. However, studies evaluating the adequacy of volume replacement therapy using BIA and its clinical usefulness in patients after major operations are limited. Thus, the aim of this study was to determine relationships between changes in values of various BIA parameters and response of critically ill patients to fluid therapy during SICU stay after surgeries. We also assessed that BIA parameters associated with volume status could correlate with clinical outcomes and the cut-off values of these parameters were also determined.

## Subjects and methods

### Subjects

**Figure 1:**
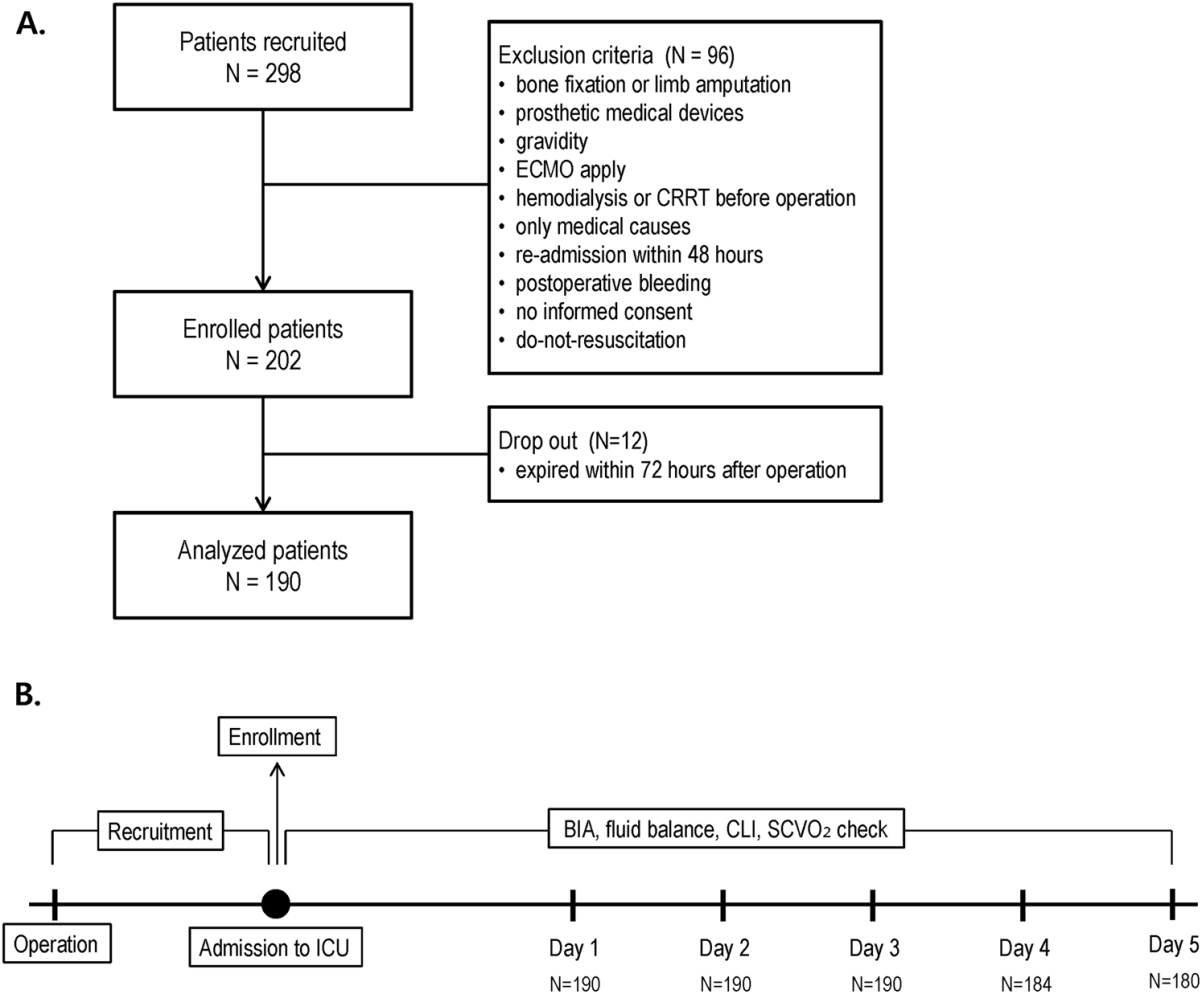
(**A**) Flow chart of patient enrollment, (**B**) flow diagram of study design.

This is a prospective observational study performed in 22-bed SICU of a single tertiary academic teaching hospital over the period from May 2019 to April 2020. Written informed consent was obtained from each patient. All patients aged over 18 years who were admitted to the SICU after operation under general anesthesia were eligible for inclusion regardless of the type of surgery. Patients who met any one of those findings same as follows were excluded from study enrollment: (1) those who had bone fixation implants or limb amputation, (2) those who had any prosthetic medical devices such as pacemakers or metallic intravascular device, (3) those who had gravidity, (4) those who underwent extracorporeal membrane oxygenation treatment (ECMO) before surgery, (5) those whose length of ICU stay was less than 48 h, (6) those who had received hemodialysis for end stage renal disease before surgery, (7) those who were readmitted within 48 h after discharge from the ICU, (8) those who were admitted to the SICU only for medical causes without operation, (9) those who were developed hypovolemic shock resulted from postoperative bleeding, or (10) those who provided informed consent for do-not-resuscitate. Patients who died within 72 h after surgery were also excluded from analysis. The flow chart about the patient selection of this study was represented in Fig. 1A. This study was approved and carefully monitored by our Institutional Review Board of the College of Medicine of the Catholic University of Korea (No. IRB; KC19RESI0584). For research involving human participants for our study, we confirmed that participants who provided informed consents. Also, this research was performed in accordance with the 1964 Declaration of Helsinki and its later amendments.

### BIA measurement

For each enrolled participant, body composition status was assessed using a commercial portable BIA device with 50-kHz alternating current (InBody S10^®^, InBody Corp., Seoul, Korea)^4^. This InBody S10 analyzer was designed for patients older than 3 years using adhesive-type or touch-type electrodes attached to four limbs. After patients were admitted postoperatively to the SICU, BIA measurements were performed daily for five days every morning (from Day 1 to Day 5) as described in the study of Lee et al.^4^ Fig. 1B was showed the flow diagram for the study design and process. The ICU is specially controlled to maintain a relatively constant and even environment, and the SICU of our institution keeps the temperature at 24 °C and the humidity between 35 to 40%. To prevent measurement error due to inappropriate posture or electrode attachment, BIA measurements were performed by one well-trained physician and supervised by one senior physician. The time required for BIA measurement was about 2 min for each person, and the data of body composition status were immediately analyzed then the result sheet was printed in real time. For each participant, the following parameters were obtained: extracellular water (ECW), intracellular water (ICW), total body water (TBW) in liters (L), ECW ratio, skeletal muscle mass, whole-body and segmental phase angle, impedance, and reactance. ECW ratio was defined as the ratio of ECW to TBW (ECW/TBW). Phase angle was defined as the physiological index of cell membrane integrity and vitality to access the quality and quantity of soft tissues and it was calculated with the following formula:

$$\emptyset = {\text{ arctan }}({57}.{296} \times {\text{Xc}}/{\text{R}})$$

($\mathrm{\varnothing }$ is phase angle; Xc is reactance; R is the resistance).

Generally, a higher value of phase angle indicates greater cellularity, cell membrane integrity, and cellular function^8–10^.

### Volume assessment

As a means to evaluate static parameters of volume status, central venous oxygen saturation (SCVO_2_) and capillary leak index (CLI) was measured every morning during intensive care unit (ICU) stay for each patient. CLI was defined as C-reactive protein over serum albumin ratio which was then multiplied by 100 as described in Malbrain et al.^11^. Daily fluid balance was defined as the difference between daily total intake (including both intravenous and enteral fluid administered, blood products, and medications) and total output of fluids (including all losses through urinary, gastrointestinal, and other drainage tubes). The fluid balance represents the total intake and output of the previous day, which was measured collectively at 6 a.m. That is an intake and output from 6 a.m. the day before to 6 a.m. on the day of the measurement. For instance, the fluid balance of day 3 is the accumulated intake and output from 6 a.m. of day 2 to 6 a.m. of day 3. Urine output was hourly measured through a foley catheter. Daily fluid balance was calculated and recorded on a specifically designed document by nurses every 24 h.

### Other data variables and clinical outcome

For enrolled participants, clinical data and outcomes were prospectively collected. From electronic medical charts, the following data were reviewed and analyzed: the demographic data including age, gender, body mass index (BMI), and the disease characteristics and severity including the diagnosis of disease and severity index using Acute Physiology and Chronic Health Evaluation II (APACHE II) and sequential organ failure assessment (SOFA) score at ICU admission, and the presence of shock. Regarding surgical profiles, the name of surgery, operation time, estimated blood loss, and amounts of transfusions were reviewed. During ICU stay, any specialized medical interventions such as renal replacement therapy, mechanical ventilation support, or extracorporeal membrane oxygenation were closely monitored and recorded. Any development of complications such as pneumonia, pulmonary edema, and acute kidney injury were monitored. Postoperative complications were classified and defined as the occurrence of morbidities above grade III using the Clavien–Dindo classification^12^. According to this classification system, Grade I complication means any deviation from the normal postoperative course without the need of any pharmacologic treatment or interventions. Grade II complications are defined as the cases that require pharmacologic treatment, total parenteral nutrition, or blood transfusion. Grade III complications are any cases that require endoscopic, radiologic, or surgical interventions. Grade IV complications are defined as life-threatening morbidities, and Grade V complications represent the death of a patient. ICU mortality or in-hospital mortality was defined as the death occurring during ICU stay or during the hospitalization period, respectively.

### Statistical analysis

All statistical analyses were conducted using SPSS statistical package software for Windows (version 21.0; SPSS Inc., Chicago, IL, USA). Continuous variables were tested for normality with the Kolmogorov–Smirnov test. Data are presented as mean ± standard deviation (SD). Overall differences were tested with Student’s t-test or ANOVA. Categorical variables are presented as proportions. They were analyzed using Chi-square (χ^2^) test or Fisher’s exact test. A *p*-value of less than 0.05 was considered statistically significant. According to the result of previous study^13–15^, the ECW ratio of more than 0.390 was defined as the overhydration group, and the patients were compared for each day dividing by the overhydration group (OH) and the normohydration group (NH). Difference in clinical outcome was analyzed according to the volume status classified by the ECW ratio on Days 1, 3, and 5, respectively. The univariate logistic regression analyses were performed to examine the predisposing factors of postoperative complications and in-hospital morality. Based on these, significantly correlated variables were analyzed using multivariate logistic regression analyses. The cut-off level of each parameter for predicting postoperative complications or in-hospital mortality was established using receiver-operating characteristic curve (ROC).

## Results

A total of 190 patients were included. Patient characteristics and clinical outcomes are shown in Table 1. Mean age of patients was 66.7 ± 15.5 years (range 18–97 years). There were 113 (59.5%) males and 77 (40.5%) females. Based on the patients' BMI data, 11.1% were underweight, 60% were normal weight, 23.1% were overweight, and 5.8% of the participants were obese. Also, subjective global assessment score for evaluating nutritional status of the participants at the time of enrollment was collected, and the mean value of that score was 1.9. 77.4% of the patients underwent the open surgery, and 16.8% of the patients had the emergency operations. The patients with estimated blood loss between 300 and 1000 ml during surgery were 151 (79.5%), those with minimal bleeding of less than 100 ml were 16 (8.4%), and those with massive bleeding of 1500 ml or more were 11 (5.8%). The amount of estimated blood loss of the participants was checked the normality and followed a normal distribution. Lengths of ICU stay and hospital stay were 6 ± 7.1 days and 24.3 ± 18.7 days, respectively. Patients with shock status accounted for 17.9%. ICU mortality and in-hospital mortality were 5.3% and 20.5%, respectively (Table 1).

**Table 1:** Demographic characteristics and clinical outcomes of study population.

Variables	N = 190 [range or %, (median)]
**Demographic characteristics**	
Age, years	66.7 ± 15.5 [18–97, (67)]
**Gender**	
Male/female	113/77 (59.5/40.5)
**BMI**	
Underweight	21 (11.1)
Normal	114 (60.0)
Overweight	44 (23.1)
Obese	11 (5.8)
Subjective global assessment	1.9 [2]
APACHE II score at ICU admission	14.3 ± 7 [0–38, (13)]
SOFA score at ICU admission	3.5 ± 3.2 [0–21, (3)]
Hypertension	81 (42.6)
Diabetes	51 (26.8)
Chronic obstructive pulmonary disease	10 (5.3)
Heart failure	9 (4.7)
End-stage liver disease	4 (2.1)
End-stage renal disease	14 (7.4)
Malignancy	83 (43.7)
**Operative profiles**	
**Type of surgery**	
Abdomen	112 (58.9)
Thoracic	36 (18.9)
Extemity	40 (21.1)
Head	6 (3.2)
Trauma	5 (2.6)
Minimal invasive surgery	43 (22.6)
Open surgery	147 (77.4)
Emergency operation	32 (16.8)
Operative time, min	205.3 ± 158.4 [95–865, (185)]
Estimated blood loss, ml	366 ± 798.7 [50–5500, (260)]
**Clinical outcomes**	
Presence of shock	34 (17.9)
Mechanical ventilation	36 (18.9)
Continuous renal replacement therapy or hemodialysis	19 (10)
Extracorporeal membrane oxygenation	2 (1.1)
Length of ICU stay, day	6 ± 7.1 [1–78, (5)]
Length of hospital stay, day	24.3 ± 18.7 [3–94, (18)]
ICU mortality	10 (5.3)
In-hospital mortality	39 (20.5)

**Figure 2:**
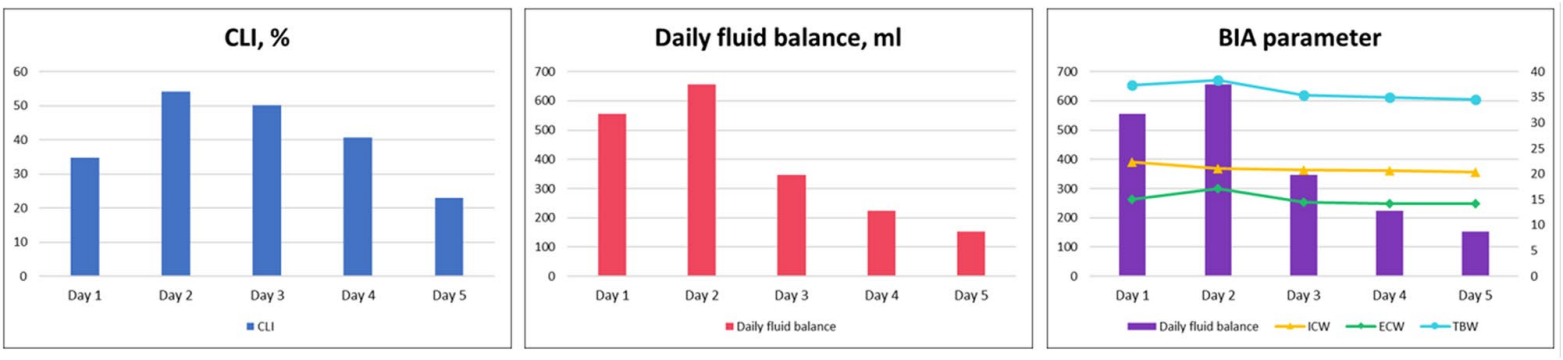
Daily variations of each volumetric variable during the first 5 days after surgery.

**Figure 3:**
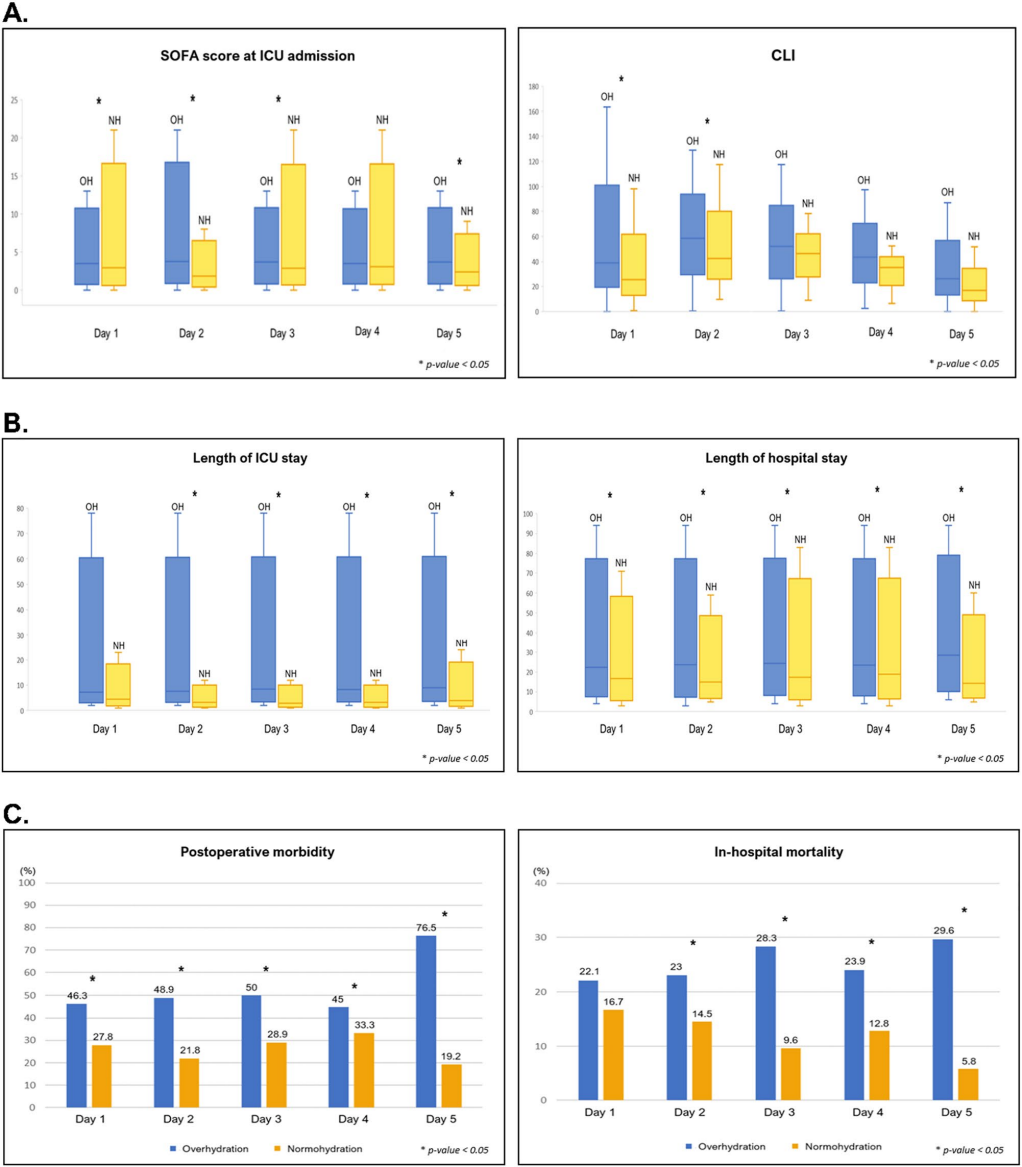
Differences in (**A**) conventional parameters such as SOFA score and CLI, (**B**) length of ICU stay and hospitalization, (**C**) postoperative outcomes such as postoperative morbidity and in-hospital mortality between overhydration group (OH) and normohydration group (NH) during the first 5 days after surgery.

Daily values of volume assessment for 5 days are shown in Table 2 and Fig. 2. CLI, daily fluid balance, and ECW ratio had the highest values on Day 2, showing a decreasing trend after that. The number of patients with overhydration status was the highest on Day 1 at 136 (71.6%) and Day 2 at 135 (71.1%), followed by a decline to 55.8% on Day 3. Comparable analyses between OH and NH on Days 1, 3, and 5 are shown in Fig. 3. On Days 3 and 5, SOFA score at ICU admission, postoperative morbidity, and length of hospital stay, length of ICU stay, ICU mortality, and in-hospital mortality were significantly higher in OH than in NH. In the univariate analysis comparing postoperative complications and mortality, the ECW ratio > 0.390 on the postoperative days 1 and 2 did not show any significant results but were only derived significantly on the 3rd day after surgery. On the other hand, the ECW ratios on the days 1 and 2, which are continuous values, were significant.

**Table 2:** Outcome of volume assessment and BID exam of study population during first 5 days after surgery.

Study day [mean ± SD, (median)]	Day 1	Day 2	Day 3	Day 4	Day 5
**Volume assessment**					
CLI^a^,%	34.8 ± 33.6 (30.7)	54.2 ± 30.5 (52.1)	50.2 ± 23.9 (48)	40.6 ± 20.2 (40.2)	23 ± 22 (22)
SCVO_2_%^b^	77.2 ± 10.1 (78.6)	75.3 ± 7.5 (75.2)	71.7 ± 6 (72.2)	74 ± 6.9 (74.5)	75.7 ± 8.5 (74.1)
Daily fluid balance^c^, ml	554.3 ± 996.6 (510)	656.5 ± 1021.5 (667)	346.5 ± 1216 (469.5)	224.5 ± 796.9 (456)	152.1 ± 998.9 (226.5)
**BID data**					
Intracellular water (ICW)	22.4 ± 21 (20.7)	21.1 ± 4.3 (21.1)	20.8 ± 4.5 (20.7)	20.7 ± 3.9 (20.5)	20.4 ± 4.6 (20.5)
Extracellular water (ECW)	15 ± 14.1 (14.5)	17.2 ± 3 (17.1)	14.5 ± 3.2 (14.4)	14.2 ± 3.2 (14.3)	14.2 ± 3.2 (14.6)
Total body water (TBW)	37.4 ± 35.1 (35.7)	38.3 ± 7 (37.7)	35.4 ± 7.4 (34.9)	34.9 ± 6.8 (35.3)	34.6 ± 7.8 (35.3)
ECW ratio (ECW/TBW)	0.4 ± 0.19 (0.35)	0.46 ± 0.03 (0.45)	0.41 ± 0.03 (0.42)	0.39 ± 0.01 (0.39)	0.4 ± 0.01 (0.4)
Overhydration^d^	136 (71.6)	135 (71.1)	106 (55.8)	109 (59.2)	81 (45)
Phase angle AT 50 kHz	3.8 ± 3.42 (3.9)	4.11 ± 1.47 (4)	3.75 ± 1.25 (3.72)	3.67 ± 1.22 (3.6)	3.57 ± 1.06 (3.5)

^a^Capillary leak index was defined as C-reactive protein (CRP) over serum albumin ratio which was multiplied by 100, as described in Malbrain et al.^11^.

^b^Central venous oxygen saturation (SCVO_2_) was measured via indwelling central line of the patients.

^c^Daily fluid balance was defined as the difference between daily total intake including both intravenous and enteral fluid administered, blood products or medications and total output including all losses through urinary, gastrointestinal, or other drainage tubes.

^d^Overhydration is defined as the case where the value of ECW ratio (ECW/TBW) is above 0.390^13–15^.

**Figure 4:**
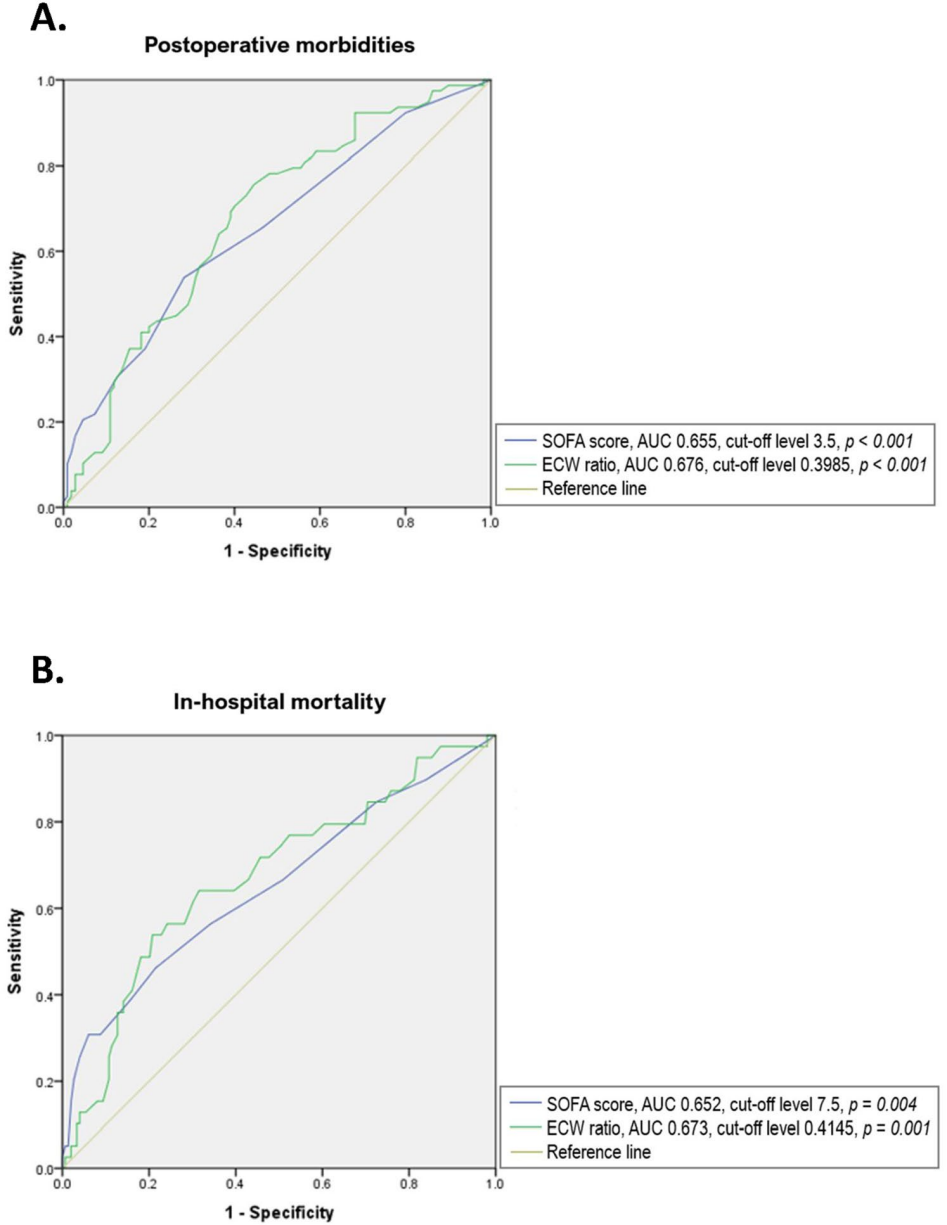
Receiver operating characteristic (ROC) curve analysis establishing cut-off values of SOFA score at ICU admission and ECW ratio at Day 3 to predict the occurrence of (**A**) postoperative morbidities and (**B**) in-hospital mortality.

Results of multivariate analysis to identify predisposing factors of postoperative morbidity occurrence and in-hospital mortality are presented in Table 3A,B. Postoperative morbidity [odds ratio (OR) 1.163, 95% CI 1.038–1.304, *p* = 0.009] and in-hospital mortality (OR 3.151, 95% CI 1.474–6.737, *p* = 0.003) were significantly more likely to occur in patients with higher SOFA scores at ICU admission. Postoperative morbidity (OR 1.182, 95% CI 1.056–1.323, *p* = 0.004) and in-hospital mortality (OR 2.040, 95% CI 1.007–4.590, *p* = 0.045) were more likely to occur in the group of patients with overhydration status on Day 3. For predicting postoperative morbidities, cut-off levels of SOFA score at admission and ECW ratio at Day 3 estimated by ROC curve were 3.5 [area under curve (AUC): 0.655; sensitivity, 53.8%; specificity, 71.4%; *p-value* < 0.001] and 0.3985 (AUC: 0.676; sensitivity, 75.6%; specificity, 55.5%; *p-value* < 0.001), respectively. (Fig. 4A). In terms of predicting of in-hospital mortality, cut-off levels of SOFA score at admission and ECW ratio at Day 3 estimated by ROC curve were 7.5 (AUC 0.652; sensitivity, 30.8%; specificity, 99.4%; *p-value* = 0.004) and 0.4145 (AUC 0.673; sensitivity, 58.3%; specificity, 79.2%; *p-value* = 0.001), respectively (Fig. 4B).

**Table 3:** Multivariate analyses of independent risk factors for the occurrence of (A) postoperative morbidities and (B) in-hospital mortality.

Variable	Odds ratio	95% CI	*p-value*
**(A) Postoperative morbidities**			
ECW ratio at Day 1	1.161 × 10^7^	0.001–1.150 × 10^17^	0.166
ECW ratio at Day 2	1.961	0.000–3.5 × 10^7^	0.937
SOFA score at ICU admission	1.163	1.038–1.304	0.009
Overhydration at Day 3	3.151	1.474–6.737	0.003
Overhydration^a^ at Day 5	1.814	0.682–4.825	0.233
**(B) In-hospital mortality**			
ECW ratio at Day 1	119.081	0.000–2.643 × 10^13^	0.720
ECW ratio at Day 2	154.035	0.000–8.814 × 10^10^	0.624
SOFA score at ICU admission	1.182	1.056–1.323	0.004
Overhydration^a^ at Day 3	2.040	1.007–4.590	0.045

Postoperative morbidities was defined as the occurrence of morbidities as grade III or more according to the Clavien–Dindo classification^12^; Grade I complication means any deviation from the normal postoperative course without the need of any pharmacologic treatment or interventions, Grade II complications are defined as the cases that require pharmacologic treatment, total parenteral nutrition, or blood transfusion, Grade III complications are any cases that require endoscopic, radiologic, or surgical interventions, Grade IV complications are defined as life-threatening morbidities, Grade V complications represent the death of a patient.

^a^Overhydration is defined as the case where the value of ECW ratio (ECW/TBW) is above 0.390^13–15^.

## Discussion

This study shows that the overhydration level of most ICU patients after surgery usually reaches its peak on Day 2 of ICU care. Their volume status then tends to decrease back to normal levels within five days. Multivariate analysis showed that the ECW ratio on Day 3 among various BIA factors has strongest association with postoperative morbidities and in-hospital mortality with cut-off values of 0.3985 and 0.4145, respectively.

For postoperative patients after having major surgeries, fluid imbalance is often observed right after surgeries due to massive transfusion and applying fluid as the means of resuscitation to correct the loss of body fluid. Fluctuation and imbalance of volume status are most remarkable in the acute phase of postoperative patient with easy-changing hemodynamics. An excessive effort of readjusting fluid imbalance and replenishing it can result in a shift of fluid into the extracellular space through injured tissues or vessels, which could in turn cause lung complications such as pulmonary edema and dyspnea^11^. This might also lead to a decline of oxygenation of tissues and tissue edema that could culminate in cell necrosis or apoptosis as possible cause of delayed wound healing and instability of anastomosis. On the other hand, the lack of sufficient replacement of fluid in acute phase cause problems such as insufficient tissue perfusion and impaired organ functions including acute kidney injury, hepatic dysfunction, or decreased bowel motility. Thus, it is important to maintain the balance of a patient’s volume status by properly assuming the amount of fluid needed in order to improve the prognosis^16^.

Actually, a very acute postoperative phase such as the first 24 h right after surgery is the period when the loss of interstitial or intravascular fluid is common due to direct consequences of surgeries. During this period, adequate tissue perfusion through fluid resuscitation is important and the positive fluid imbalance observed in this period would be considered a natural course, not relevant to poor prognosis^17,18^. It might be more important to examine a patient’s volume status and prevent problems due to unnecessary fluid overloading or restriction in the acute phase at 48–72 h after major surgery rather than in the very acute phase. At the acute phase, previous oliguria caused by secretion of stress hormone and increase in renal reabsorption are usually improved followed by progression to a diuretic phase. Colin et al.^19^ and Boerma et al.^20^ have reported that on the third day of shock, microcirculatory blood flow begins to back to normal and the homeostasis of cytokines effective in healing microcirculatory disruptions begins to work, subsequently leading to the closure of the capillary leak. Therefore, authors suppose that the positive fluid imbalance observed in the period of 48–72 h after surgery is related to organ dysfunctions and thus relevant to the prognosis.

In this study, the ECW ratio of postoperative patients turned out to be the highest on the second day of ICU admission, and then it tended to decrease. The ECW ratio refers to the proportion of extracellular fluid and it is known to reflect edematous status and malnutrition in patients^15^. We divided patients into two groups, OH and NH, according to their ECW ratio and identified a significant difference in outcomes between two groups. This finding implies the feasibility of ECW ratio as a tool to assess volume status of a patient in the acute phase after surgery. ECW ratios higher than 0.3985 and 0.4145 on the third day of ICU could be a predisposing factors of postoperative complications and in-hospital mortality, respectively. This supports our conclusion that a proper volume status management in the acute postoperative phase would have clinical importance.

Additionally, the measurement using BIA usually takes less than two minutes as a non-invasive approach with a portable machine is suitable for an immobilized patient^4,21,22^. It could be also used safely for postoperative critical ill patients with poor clinical conditions such as bleeding without giving them time-related discomfort or any other risks of procedure. Actually, there were no complication or discomfort of patients relevant to the BIA use in our study. Authors suppose that BIA could be helpful for measuring the volume status of a critical ill patient, especially in an acute phase for patients who suffer from severe pain due to operative wounds or multiple drains that are too serious to take repeated invasive examinations.

As shown in Fig. 2, changes in various BIA parameters had similar patterns with those of daily fluid balance as well as those with CLI values. CLI is associated with increased vascular permeability that could result from inflammation and shock. Inflammation can lead to releases of pro-inflammatory cytokines and stress hormones that would consequently result in vascular permeability increase, and transcapillary albumin leak. It can cause the impaired regional tissue oxygenation, enhanced compensatory neuroendocrine reflexes, and increasing fluid retention of the kidney that eventually cause a positive fluid balance^23^. These events can increase CLI levels consecutively. For a patient after a major surgery whose physiologic state is similar to those in a shock status, the capillary leak and tissue edema are common. A previous research^19,20^ has reported that CLI levels of patients tend to increase until a crucial turning point is observed on the third day of shock. After the third day of shock, their microcirculatory blood flows begin to be normalized accompanied by the closure of capillary leak. This can explain our findings that CLI levels began to decrease after the third day and that CLI level was highly correlated with ECW ratio on BIA. Excessive increase of interstitial volume due to this capillary leak could lead to pulmonary edema and contribute to the occurrence of lung complications of fluid overload^24^. Although this study failed to analyze the direct relation with respiratory complication, the authors believe that an elevated ECW ratio and overhydration status on Day 3 after operation can cause a burden for respiratory functions. Especially, patients after major abdominal surgeries are often vulnerable to respiratory distress because proper deep breathing and expectoration are very difficult due to surgical wound, pain, or residual effect of general anesthesia. Therefore, we expect that an overhydration status of a patient as ECW ratio on postoperative day 3 more than 0.3985 might have a risk of respiratory complications, requiring the efforts to properly readjust the fluid administration and volume status. To verify this assumption, additional studies with large sized data collection and analysis are needed.

Despite these interesting outcomes, results of the current study should be interpreted with caution due to various limitations. Firstly, our data analysis had some bias due to its observational study design of a single center despite of our strict enrollment and exclusion criteria. The patients with CRRT or ECMO treatment in Table 1 represented newly applied patients during postoperative period of ICU care. The patients receiving treatments such as ECMO and CRRT due to changes in condition caused by postoperative complications have different volume status in their body for fluid management compared to patients who do not receive these treatments. To reduce the bias in BIA measurements on the volume status of this patient group, subsequent studies on large patient enrollment and subgroup analysis would be needed. Most of the enrolled patients had elective surgery rather than emergency operation, and few patients needed the vaso-active drugs after surgery. Most patients with hemodynamic instability had undergone the emergency operation, and were able to recover with the fluid resuscitation. When vaso-active drugs were required because it was difficult to recover from the hemodynamic condition only with fluid resuscitation, norepinephrine was used as the first-choice vaso-active drug according to the SSC guideline. It is difficult to completely rule out that the use and type of vaso-active drugs may act as a bias factor for BIA results. According to the recent papers published so far^16,18^, there are several treatment principles for fluid treatment, but the amount of the fluid given and timing of the administration are debatable in the following study, it is necessary to prospectively apply different strategies of fluid management according to randomized designs and perform a subgroup analysis to determine whether there are any differences in clinical outcomes by measuring the BIA for each case. Additionally, various factors of ICU environment such as ambient air, skin temperature, seating, and specific conductance of ICU bed that might affect BIA measurements could not be understood. Secondly, we did not perform subgroup analysis according to the type of shock or surgery mainly due to small numbers of participants. Most of the enrolled patients of this study received abdominal surgery due to characteristics of our SICU. But, there were relatively few patients who underwent vascular surgery or trauma. The patients who had abdominal surgery had higher systemic inflammation, extensive organ injury, and tissue damage, and would be expected to have greater BIA changes with fluid management compared to the patients who underwent endovascular surgery or operation of extremities. The small number of participants could be also a reason for failure to confirm the statistical significance of overhydration status in the prediction of occurrence of pulmonary complications. A well-designed prospective randomized study involving a large number of participants should be conducted in the near future to confirm results of the current study. Thirdly, since a high ECW ratio means fluid overload as well as when the body cell mass is low, it is difficult to distinguish the two using only BIA data. However, the results of this study are determined to be changes in the ECW ratio due to fluid overload, but it cannot be completely excluded that it may be related to malnutrition and low body cell mass in patients. Moreover, in this study, the baseline BIA were not measured before surgery, but were measured immediately after entering the SICU after surgery at the time of enrollment, so it is difficult to compare the changes in BIA data before and after surgery. In particular, in the patients who have underlying diseases such as chronic kidney disease, heart failure, and chronic obstructive pulmonary disease with poor cell membrane conditions, there may be limitations in interpreting the fluid overhydration status only with BIA data after surgery. The phase angle of the BIA parameters is an indicator that reflects the health condition of cells or cell membranes and can be used as a nutritional indicator. There is no baseline phase angle data, so the use of the phase angles in comparing changes in the nutritional condition of patients before and after surgery is also limited, either. Therefore, the results of baseline data before surgery should be included to overcome these limitations and ensure more clear comparisons in the near future study.

Nevertheless, this study is meaningful in that it differs from existing studies. It was a prospective cohort study conducted on patients after major operation. Also, in the current study, SICU patients in an acute phase were observed intensively. BIA is a non-invasive method that is easy to operate and measure and it is expected to provide a foundation of the fluid management guideline in acute phase of ICU patients after major operations. Monitoring fluid imbalance through BIA could prevent the occurrence of complications related fluid imbalance earlier, eventually improving a patient’s clinical outcome.

In conclusion, the use of BIA to estimate volume status is a feasible method that is easy, safe, and adaptable for critical patients in immobilization status after operations. We found that an overhydration status with ECW ratio > 0.390 on Day 3 after operation was related to postoperative morbidity and in-hospital mortality occurrence.

## Abbreviations

APACHE II: Acute physiology and chronic health evaluation II
AUC: Area under curve
BIA: Biochemical impedance analysis
BMI: Body mass index
CLI: Capillary leak index
CRRT: Continuous renal replacement therapy
ECMO: Extracorporeal membrane oxygenation treatment
ECW: Extracellular water
ICW: Intracellular water
NH: Normohydration group
OH: Overhydration group
OR: Odds ratio
ROC: Receiver operating characteristic curve
SCVO_2_: Central venous oxygen saturation
SICU: Surgical intensive care unit
SOFA: Sequential organ failure assessment
TBW: Total body water

## References

[1] Zhang Z, Ho KM, Hong Y (2019). Machine learning for the prediction of volume responsiveness in patients with oliguric acute kidney injury in critical care. Crit. Care..

[2] Bloos F, Zhang Z, Boulain T (2016). Lactate-guided resuscitation saves lives: Yes. Intensive Care Med..

[3] Gu WJ, Zhang Z, Bakker J (2015). Early lactate clearance-guided therapy in patients with sepsis: A meta-analysis with trial sequential analysis of randomized controlled trials. Intensive Care Med..

[4] Lee YH, Lee JD, Kang DR, Hong J, Lee JM (2017). Bioelectrical impedance analysis values as markers to predict severity in critically ill patients. J. Crit. Care.

[5] Yilmaz Z, Yildirim Y, Aydin FY, Aydin E, Kadiroglu AK, Yilmaz ME (2014). Evaluation of fluid status related parameters in hemodialysis and peritoneal dialysis patients: Clinical usefulness of bioimpedance analysis. Medicina (Kaunas)..

[6] Basso F, Berdin G, Virzi GM, Mason G, Piccinni P, Day S (2013). Fluid management in the intensive care unit: Bioelectrical impedance vector analysis as a tool to assess hydration status and optimal fluid balance in critically ill patients. Blood Purif..

[7] Buffa R, Mereu E, Comandini O, Ibanez ME, Marini E (2014). Bioelectrical impedance vector analysis (BIVA) for the assessment of two-compartment body composition. Eur. J. Clin. Nutr..

[8] Barbosa-Silva MCG, Barros AJD (2005). Bioelectric impedance and individual characteristics as prognostic factors for post-operative complications. Clin. Nutr..

[9] Lukaski HC, Kyle UG, Kondrup J (2017). Assessment of adult malnutrition and prognosis with bioelectrical impedance analysis: Phase angle and impedance ratio. Curr. Opin. Clin. Nutr. Metab. Care.

[10] Stapel SN, Looijaard WGPM, Dekker IM, Girbes ARJ, Weijs PJM, Straaten HMOV (2018). Bioelectrical impedance analysis-derived phase angle at admission as a predictor of 90-day mortality in intensive care patients. Eur. J. Clin. Nutr..

[11] Cordemans C, De Laet I, Van Regenmortel N, Schoonheydt K, Dits H, Huber W (2012). Fluid management in critically ill patients: The role of extravascular lung water, abdominal hypertension, capillary leak, and fluid balance. Ann Intensive Care..

[12] Dindo D, Demartines N, Clavien PA (2004). Classification of surgical complications: A new proposal with evaluation in a cohort of 6336 patients and results of a survey. Ann. Surg..

[13] Noda Y, Suzuki H, Kanai T, Samejima Y, Nasu S, Tanaka A (2020). The association between extracellular water-to-total body water ratio and therapeutic durability for advanced lung cancer. Anticancer Res..

[14] Nishikawa H, Yoh K, Enomoto H, Ishii N, Iwata Y, Nakano C (2018). Extracellular water to total body water ratio in viral liver diseases: A study using bioimpedance analysis. Nutrients.

[15] Park KH, Shin JH, Hwang JH, Kim SH (2017). Utility of volume assessment using bioelectrical impedance analysis in critically ill patients receiving continuous renal replacement therapy: A prospective observational study. Korean J. Crit. Care Med..

[16] Kayilioglu SI, Dinc T, Sozen I, Bostanoglu A, Cete M, Coskun F (2015). Postoperative fluid management. World J. Crit. Care Med..

[17] Malbrain M, Van Regenmortel N, Saugel B, De Tavernier B, Van Gaal PJ, Joannes-Boyau O (2018). Principles of fluid management and stewardship in septic shock: It is time to consider the four D's and the four phases of fluid therapy. Ann. Intensive Care..

[18] Silversides JA, Major E, Ferguson AJ, Mann EE, McAuley DF, Marshall JC (2017). Conservative fluid management or deresuscitation for patients with sepsis or acute respiratory distress syndrome following the resuscitation phase of critical illness: A systematic review and meta-analysis. Intensive Care Med..

[19] Rivers EP (2006). Fluid-management strategies in acute lung injury–liberal, conservative, or both?. N. Engl. J. Med..

[20] Boerma EC, van der Voort PHJ, Spronk PE, Ince C (2007). Relationship between sublingual and intestinal micro circulatory perfusion in patients with abdominal sepsis. Crit. Care Med..

[21] Malbrain ML, Huygh J, Dabrowski W, De Waele JJ, Staelens A, Wauters J (2014). The use of bio-electrical impedance analysis (BIA) to guide fluid management, resuscitation and deresuscitation in critically ill patients: A bench-to-bedside review. Anaesthesiol. Intensive Ther..

[22] Denneman N, Hessels L, Broens B, Gjaltema J, Stapel SN, Stohlmann J (2020). Fluid balance and phase angle as assessed by bioelectrical impedance analysis in critically ill patients: A multicenter prospective cohort study. Eur. J. Clin. Nutr..

[23] Fleck A, Raines G, Hawker F, Trotter J, Wallace PI, Ledingham IM (1985). Increased vascular permeability: A major cause of hypoalbuminaemia in disease and injury. Lancet.

[24] Schrier RW, Wang W (2004). Acute renal failure and sepsis. N. Engl. J. Med..

